# Involvement of CCL2 in Salivary Gland Response to Hyperosmolar Stress Related to Sjögren’s Syndrome

**DOI:** 10.3390/ijms25020915

**Published:** 2024-01-11

**Authors:** Clara Chivasso, Dorian Parisis, Xavier Cabrol, Azine Datlibagi, Valérie Delforge, Françoise Gregoire, Nargis Bolaky, Muhammad Shahnawaz Soyfoo, Jason Perret, Christine Delporte

**Affiliations:** 1 Laboratory of Pathophysiological and Nutritional Biochemistry, Université Libre de Bruxelles, 1070 Brussels, Belgium; clara.chivasso@hubruxelles.be (C.C.); dorian.parisis@ulb.be (D.P.); azine.datlibagi@ulb.be (A.D.); valerie.delforge@ulb.be (V.D.); francoise.gregoire@ulb.be (F.G.); nargis.bolaky@ulb.be (N.B.); jason.perret@ulb.be (J.P.); 2 Department of Rheumatology, The Brussels University Hospital—Erasme Hospital, Université Libre de Bruxelles, 1070 Brussels, Belgium; xavier.cabrol@ulb.be (X.C.); muhammad.shah.soyfoo@ulb.be (M.S.S.)

**Keywords:** hyperosmotic stress, salivary gland epithelial cells, CCL2, inflammation, Sjögren’s syndrome

## Abstract

In primary Sjögren’s syndrome (pSS) patients, salivary gland (SG) epithelial cells (SGECs) could be exposed to chronic hyperosmotic stress (HOS), consecutive to their destruction and deregulation, that exacerbates an inflammatory response. The aims of this study were to assess the mechanism accounting for C-C motif chemokine ligand 2 (CCL2) expression in an immortalized human salivary gland epithelial acinar cell line (NS-SV-AC) subjected to HOS, as well as the involvement of CCL2 in pSS. CCL2 mRNA and protein levels were determined via RT-qPCR and ELISA. Reporter plasmids and a promoter pull-down assay were used to identify transcription factors associated with CCL2 mRNA increase. Our data showed that HOS-induced CCL2 mRNA increase was independent of the nuclear factor of activated T-cells 5 (NFAT5) and nuclear factor-kappa B (NFkB) but involved Kruppel-like factor 5 (KLF5). CCL2 protein levels, quantified by enzyme-linked immunosorbent assay (ELISA) in sera samples from pSS patients, correlated with the European Alliance of Associations for Rheumatology’s Sjogren’s syndrome disease activity index (ESSDAI) score for systemic activity. In addition, CCL2 protein levels were higher in patients with biological activity, cutaneous manifestations, and ESSDAI score superior or equal to five. Our data suggest that chronic HOS could exacerbate pSS disease by contributing to the inflammatory process induced by the expression and secretion of CCL2.

## 1. Introduction

Primary Sjögren’s syndrome (pSS) is the second most common chronic autoimmune disorder, displaying a high prevalence in female patients compared to male (ratio of 9:1) [1]. The most important hallmark of SS consists in the lymphocytic infiltration of exocrine glands, in particular salivary and lacrimal glands, leading to xerostomia and keratoconjunctivitis sicca symptoms [2]. The pathophysiology of pSS is complex and multifactorial and involves epithelial cell activation and deregulation and immunological anomalies [3]. Indeed, salivary gland epithelial cells (SGECs) play a central role in the immune responses [4], as suggested by the presence of infiltrating lesions (epithelitis) [5], as well as increased expression of multiple pro-inflammatory cytokines in the histopathological lesions [6,7,8]. SGECs may act as nonprofessional antigen-presenting cells participating actively in the autoantigen presentation, as well as in the secretion of various inflammatory molecules involved in the recruitment, activation, and differentiation of immune cells [9]. Numerous molecules are involved in these processes including major histocompatibility complex (MHC) proteins class-I (HLA-ABC) and class-II (HLA-DR) [10,11], intercellular adhesion molecule-1 (CD54/ICAM1), vascular cell adhesion molecule-1 (CD106/VCAM), E-selectin [9,12], costimulatory molecules, C-C motif chemokine ligand 2 (CCL2; also termed monocyte chemoattractant protein-1 (MCP-1)), interleukin-6 (IL-6), tumor necrosis factor alpha (TNFα) [13], and B-cell differentiating factor (BAFF) [7].

Furthermore, pSS SG acinar cell apoptosis, as well as abnormal aquaporin-5 (AQP5) cellular localization (mostly basolateral instead of apical) [14,15], could promote the formation of a chronic hyperosmotic gradient resulting from reduced water flow to the acinar lumen and KHCO_3_ secretion by ductal cells, leading in turn to a chronic exposure of SGECs to HOS. Upon HOS-induced shrinkage, cells are able to regulate their volume by a mechanism called regulatory volume increase (RVI) [16]. In RVI, the first phase involves short-term transport of ions and water into the cells [16], while a second phase involves a long-term accumulation of intracellular organic osmolytes as a result of the activation of the nuclear factor of activated T-cells 5 (NFAT5) (also termed tonicity-responsive enhancer-binding protein (TonEBP) or osmotic response element-binding protein (OREBP) [17,18]. Activated NFAT5 undergoes nuclear translocation and transactivates osmoprotective genes such as aldose reductase [19,20,21], taurine transporter [22,23], and sodium/myo-inositol transporter [24]. Consequently, intracellular organic osmolytes (such as sorbitol, taurine, and myo-inositol) accumulate inside the cells and trigger water influx into the cells restoring the cell volume [24,25]. HOS can also induce additional effects in cells including DNA damage, cycle arrest, apoptosis, mitochondrial depolarization, alteration of transcriptional and translational machineries, oxidative stress, cytoskeleton rearrangement, and modulation of stress proteins [24]. In addition, prolonged HOS modifies innate and adaptive immune responses as observed in mononuclear cells subjected to HOS [26,27,28]. Several studies have shown that an increase in local salt concentration caused CD4+ T cell differentiation into Th17 [29,30] and the development of autoimmune diseases [30,31]. Although the biological mechanisms leading to SS sicca symptoms are poorly understood, increasing evidence suggests that dryness of the eyes and mouth are not only the result of SG destruction but primarily the consequences of cytokine, autoantibody, and soluble factor production exacerbating the pathological inflammatory process [3,5,32,33].

To date, several studies have shown that CCL2 is highly expressed in SS patients’ sera, tears, and saliva [34,35,36,37] and in particular in those with ectopic germinal structures in SGs [38]. CCL2 is considered as a potential target for the treatment of several autoimmune diseases. Indeed, CCR2 genetic deletion or inhibition conferred resistance to disease in a murine model of Guillain–Barré syndrome [39] and reduced immune cell infiltration and retinal damage in an autoimmune uveitis mouse model [40]. 

In a previous publication, we analyzed the transcriptome of immortalized human salivary gland acinar cells (NS-SV-AC) subjected to HOS by the addition in a culture medium of 100 mM of sodium chloride (NaCl; Na100) and 200 mM sucrose (Su200). The study revealed 1376 differentially expressed genes (DEGs) common to both Na100 and Su200 stimulations, with 1035 upregulated and 341 downregulated DEGs [41]. Among the most 150 upregulated DEGs, we identified CCL2 belonging to the enriched category of interferon (IFN)-induced genes [41]. 

In conclusion, HOS may play a pivotal role in the pathogenesis of pSS by exacerbating pro-inflammatory condition in SGECs. Considering the relevance of the inflammatory process in pSS pathogenesis, the goals of this study are to assess the mechanism responsible for CCL2 induction and the role of CCL2 in pSS. 

## 2. Results

### 2.1. NaCl Increases CCL2 Expression in Human SG Acinar Cells

**Figure 1:**
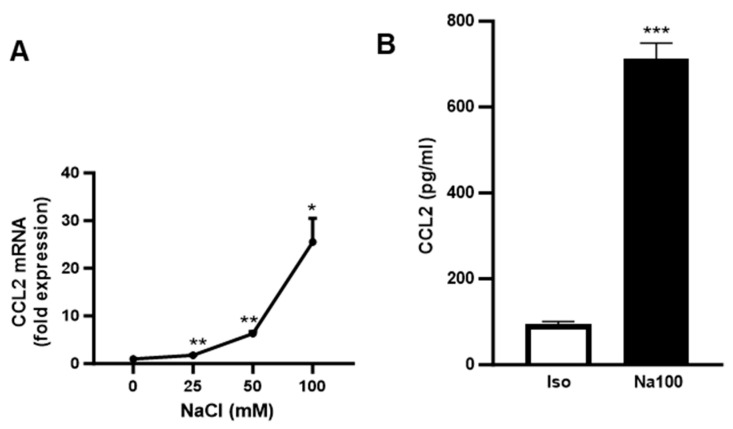
HOS induced an increase in CCL2 mRNA and protein in NS-SV-AC cells. (**A**) NS-SV-AC cells were stimulated for 8 h with 0, 25, 50, and 100 mM of NaCl. CCL2 mRNA was determined via RT-qPCR. Data are expressed as the mean ± S.E.M. of the relative mRNA over Iso, the latter being set to 1 (in fold expression), following normalization with appropriate reference genes (n = 4). (**B**) ELISA assay on NS-SV-AC cells in the absence or presence of Na100 after 24 h of stimulation. Data are expressed as the mean ± S.E.M. of pg/mL of CCL2 protein (n = 4). Data were analyzed using repeated-measures Anova followed by post hoc Dunnett tests (**A**) or paired Student *t*-test (**B**). *: *p* < 0.05, **: *p* < 0.01; ***: *p* < 0.001.

NS-SV-AC cells were incubated for 8 h or 24 h in a culture medium in the absence (Iso) or presence of supplementary increasing concentrations of NaCl (Na25, Na50, and Na100). After 8 h, CCL2 mRNA levels were significantly increased in response to increasing concentrations of NaCl (*p* = 0.0174), with a ±25-fold rise at 100 mM (Figure 1A). Furthermore, after 24 h, the quantity of CCL2 protein was significantly increased fivefold upon treatment with Na100 (Figure 1B).

### 2.2. NaCl-Induced CCL2 mRNA Is Independent of NFAT5 and NFkB Transactivation Activity

**Figure 2:**
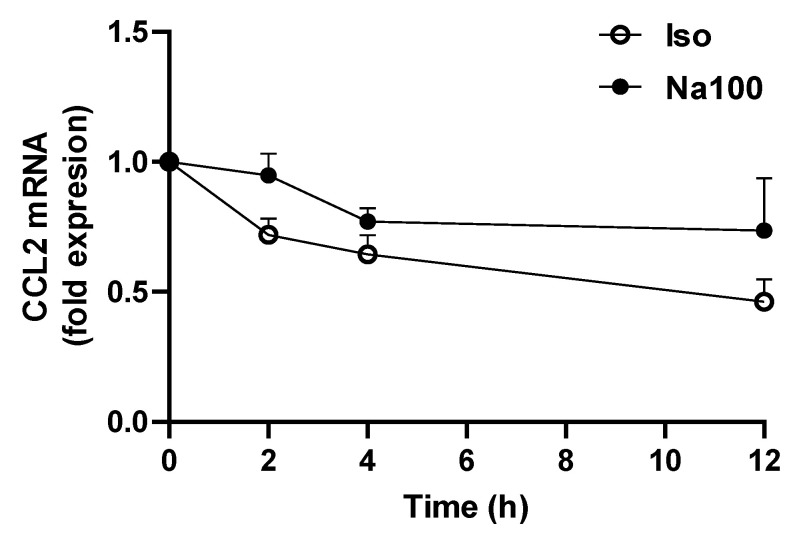
Determination of CCL2 mRNA half-life. NS-SV-AC cells were treated with Iso or Na100 for 8 h prior to incubation for various times with 1 µM actinomycin D. Data are expressed as the mean ± S.E.M. of the relative mRNA levels over Iso or Na100 at 0 h, both of the latter being set to 1 (in fold expression), following normalization with appropriate reference genes (n = 3). Data were analyzed using two-way repeated-measures Anova (*p* = 0.0284 for effect of time; *p* was non-significant for effect of treatment).

To assess whether CCL2 mRNA induction in response to Na100 was due to increased transcription or reduced mRNA degradation, the CCL2 mRNA half-life was determined in NS-SV-AC cells subjected to Iso and Na100 in the presence of actinomycin D, a transcription inhibitor. Data showed a significant effect of time (*p* = 0.0284) but not of Na100 treatment (*p* = 0.0944) on the CCL2 mRNA quantity, suggesting that Na100 did not modify the CCL2 mRNA half-life (Figure 2). These data suggest that Na100 increased CCL2 mRNA levels through transcription rather than via stabilization of CCL2 mRNA.

**Figure 3:**
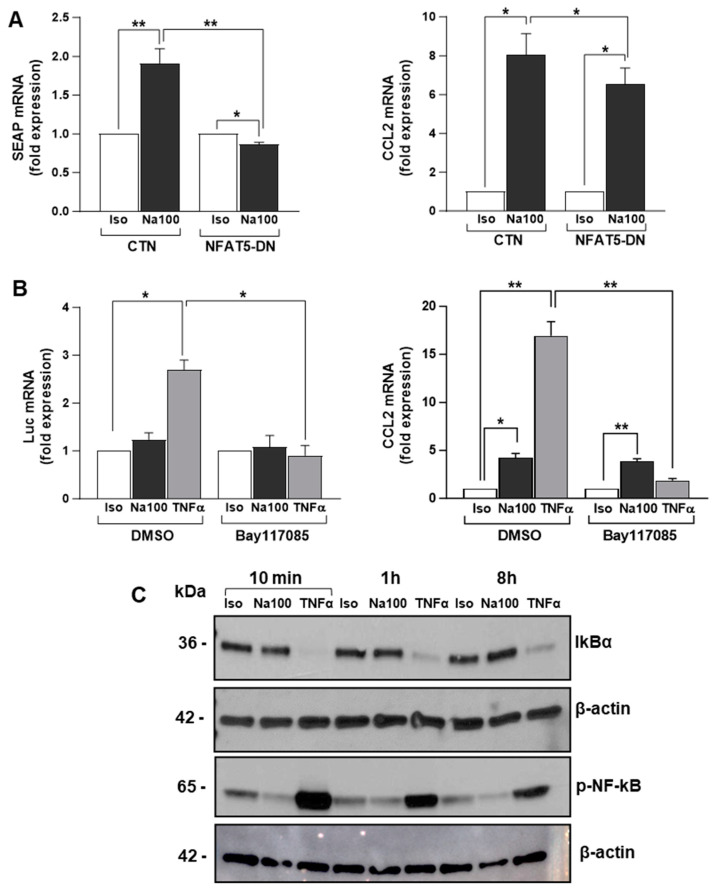
HOS-induced CCL2 mRNA increase is independent of NFAT5 and NF-ĸB activation. (**A**) NS-SV-AC cells were co-transfected with pSEAP-TonE plasmid and CTN or NFAT5-DN and then treated with Iso or Na100 for 8 h. SEAP (**left**) and CCL2 (**right**) mRNA levels were quantified by RT-qPCR. Data are expressed as the mean ± S.E.M. of the relative mRNA levels (in fold expression) over Iso, the latter being set to 1, following normalization with appropriate reference genes (n = 4). (**B**) NS-SV-AC cells were transfected with NF-ĸB-Luc plasmid. Then, 48 h post-transfection, cells were preincubated for 1 h with DMSO or Bay117085 and then subjected to Iso, Na100, and TNFα for 8 h. Luciferase (Luc) (**left**) and CCL2 (**right**) mRNA levels were quantified by RT-qPCR. Data are expressed as the mean ± S.E.M. of the relative mRNA levels (in fold expression) over Iso, the latter being set to 1, following normalization with appropriate reference genes (n = 4). (**C**) Western blot of IKBα and p-NF-ĸB (Ser536) on total proteins from NS-SV-AC cells treated with Iso, Na100, and TNFα for 10 min, 1 h, and 8 h. For (**A**,**B**): data were analyzed using a *t*-test for a unique sample and a paired *t*-test. *: *p* < 0.05, **: *p* < 0.01.

To determine the possible involvement of NFAT5 in NaCl-induced CCL2 transcription, we evaluated NFAT5 transactivation activity using an NFAT5 reporter plasmid (pSEAP-TonE) in the absence or presence of a dominant negative (DN) form of NFAT5 (NFAT5-DN) in response to Iso or Na100 (Figure 3A). In response to Na100, SEAP mRNA levels significantly increased by 1.9-fold as compared to Iso. NFAT5-DN did not modify SEAP mRNA levels measured under Iso, while it significantly abolished an Na100-induced increase in SEAP mRNA. These results indicated that HOS induced the activation of NFAT5. Then, on the same cells, we determined whether NFAT5 activation was responsible for HOS-induced CCL2 expression. However, the HOS-induced CCL2 mRNA levels remained unchanged in the presence of NFAT5-DN. Consequently, HOS-induced CCL2 transcription in NS-SV-AC cells was independent of NFAT5 transactivation activity (Figure 3A).

As NF-ĸB is well known to promote CCL2 transcription by binding to its gene promoter [42] and to be activated by hyperosmolarity [43,44], we hypothesized NF-ĸB might be responsible for HOS-induced CCL2 mRNA expression in NS-SV-AC cells. To test this hypothesis, NF-ĸB transactivation activity and CCL2 mRNA expression were determined using an NF-ĸB reporter plasmid (pNF-ĸB-Luc) and an irreversible inhibitor of IkBα phosphorylation preventing NF-ĸB activation (Bay117085). In the absence of an NF-ĸB inhibitor, TNFα (used as positive control) induced a significant 2.5-fold increase in Luc mRNA, while Na100 had no effect. In addition, Bay117085 significantly abolished the TNFα- but not the Na100-induced CCL2 mRNA expression (Figure 3B). These data were corroborated by the degradation of IKBα (consecutive to its phosphorylation) in response to TNFα, but not to Na100, and the phosphorylation of NF-ĸB (released from IKBα inhibitory activity) (Figure 3C). Taken together, these data suggest that an HOS-driven CCL2 mRNA increase does not involve NF-ĸB activation.

### 2.3. Involvement of Kruppel-like Transcription Factor 5 (KLF5) in the Na100-Induced Increase in CCL2 mRNA

**Figure 4:**
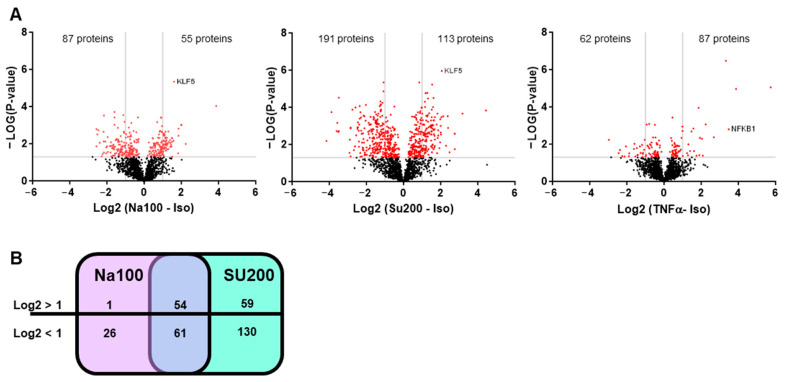
Identification of proteins differentially binding to the CCL2 gene promoter in response to HOS. (**A**) Volcano plots of proteins identified by mass spectrometry following oligonucleotide pulldown assays. Horizontal bars indicate −Log10 *p*-value, with cut-off set to 1.5; vertical bars indicate Log2 (Na100 or Su200 or TNFα -Iso), with cut-off set to −1 and +1. Red and black dots represent proteins respectively above or below the set cut-off values. (**B**) Venn diagram indicating the number of proteins with Log2 (Na100-Iso) or Log2 (Su200-Iso) >1 or <1.

An oligonucleotide pulldown assay was used to identify the transcription factors differentially binding to the CCL2 promoter region under Na100 and Su200 (used as another agent inducing HOS) as compared to Iso (used as control). Following the pulldown assay, the nuclear proteins-DNA-streptavidin agarose bead complexes were subjected to trypsin digestion and mass spectrometry analysis. To identify the proteins differentially binding to the CCL2 gene promoter, averages of label-free quantitation (LFQ) intensity values expressed in Log2 ratios were plotted. Volcano plots of CCL2-promoter-binding proteins revealed that under Na100 stimulation, 55 and 87 proteins, respectively, bound with a Log2FC superior or inferior to 1 (Figure 4A). Under Su200, 113 and 191 proteins, respectively, bound with a Log2FC superior or inferior to 1 (Figure 4A). Under TNFα, 87 and 62 proteins, respectively, bound with a Log2FC superior or inferior to 1 (Figure 4A). Venn diagram indicates that 54 and 61 proteins, respectively, bound with a Log2FC superior or inferior to 1 under both Na100 and Su200 (Figure 4B). Among the proteins with Log2FC superior to 1 under both Na100 and Su200, KLF5 was the most significant. Under TNFα stimulation (used as a technical positive control of the experiment), NFKB1 was the most significant, therefore validating the experimental settings. 

**Figure 5:**
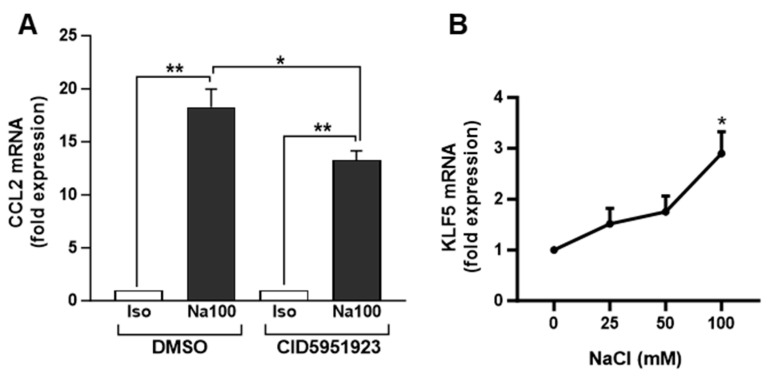
Involvement of KLF5 inhibitor in the NaCl-induced CCL2 mRNA levels. (**A**) NS-SV-AC cells were preincubated for 1 h with DMSO or 10 µM CID5951923 (diluted in DMSO) and then treated with Iso or Na100 for 8 h. CCL2 mRNA levels were quantified by RT-qPCR. Data are expressed as the mean ± S.E.M. of the relative mRNA levels (in fold expression) over Iso, the latter being set to 1, following normalization with appropriate reference genes (n = 4). Data were analyzed using *t*-test for unique sample and paired *t*-test. *: *p* < 0.05, **: *p* < 0.01. (**B**) NS-SV-AC cells stimulated during 8 h with additional 0, 25, 50, and 100 mM of NaCl in the culture medium. KLF5 mRNA levels were determined by RT-qPCR. Data are expressed as the mean ± S.E.M. of the relative mRNA levels (in fold expression) over Iso set to 1, following normalization with appropriate reference genes (n = 4). Data were analyzed using repeated-measures Anova followed by post hoc Dunnett tests. *: *p*< 0.05.

To determine the possible involvement of KLF5 in Na100-induced CCL2 transcription, we evaluated the effect of a KLF5 inhibitor (CID5951923) on the Na100-induced CCL2 expression. Our data indicated that CID5951923 inhibited by approximately 27% the Na100-induced CCL2 mRNA levels, suggesting that KLF5 is implicated in the transcription of CCL2 induced by Na100 and Su200 (Figure 5A). In addition, NaCl induced a dose-dependent increase in KLF5 mRNA levels, with an approximately threefold increase at 100 mM NaCl (Figure 5B).

### 2.4. CCL2 Secretion Correlates with ESSDAI, Biological and Cutaneous Manifestations in pSS Patients

**Figure 6:**
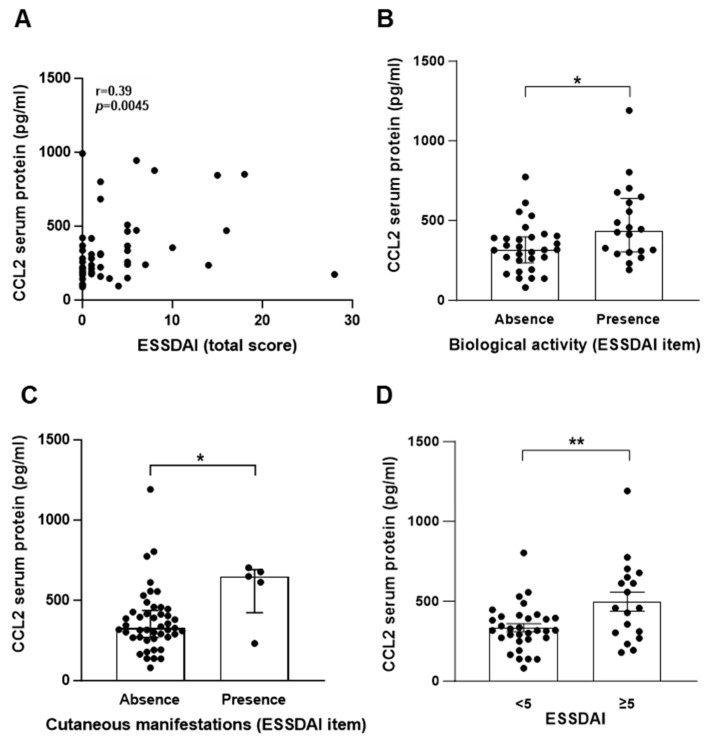
CCL2 protein level in sera from pSS patients correlates with biological manifestations and ESSDAI score. CCL2 protein was quantified by ELISA in sera from pSS patients (n = 50). (**A**) Correlation between CCL2 protein levels and ESSDAI total score. (**B**–**D**) CCL2 protein level in pSS patients with the absence or presence of biological manifestation (**B**), cutaneous manifestations (**C**), or ESSDAI score <5 or ≥5 (**D**). (**A**): data were analyzed using Spearman’s r-test; (**B**,**C**): data are expressed as the median ± interquartile range and data were analyzed using Mann–Whitney’s U-test; (**D**): data are expressed as mean ± S.E.M. and data were analyzed using unpaired *t*-test (n = 50). (**A–D**): Each dot represents data from a pSS patient; *: *p* < 0.05; **: *p*< 0.01.

To provide insight into pSS pathogenesis, we investigated the potential association between CCL2 protein levels in serum samples and the pathophysiology of pSS. To this end, we explored if variations in the systemic CCL2 concentration correlated with clinicoepidemiological parameters (described in Table 1). CCL2 protein levels correlated with the ESSDAI total score for systemic activity (*p* = 0.043; Figure 6A). The amount of CCL2 protein was significantly higher in patients with the presence of biological (*p* = 0.019; Figure 6B) and cutaneous manifestations (*p* = 0.039; Figure 6C), as defined in EULAR Sjögren’s syndrome disease activity index (ESSDAI) (clinical index designed to measure disease activity in patients with pSS [45]), than in patients with no biological manifestations. In addition, pSS patients with active disease (ESSDAI ≥ 5) have higher amounts of CCL2 protein than patients with no systemic pSS (Figure 6D, *p* = 0.005). No associations were observed between CCL2 protein levels and EULAR Sjogren’s Syndrome Patient Reported Index (ESSPRI) (clinical index designed to measure the severity of pSS symptoms), presence of sicca complex items (fatigue, chronic pain or dryness [46]), focus score, autoimmunity markers (ANA, anti-Ro/SSA positivity), and objective exocrine testing (Schirmer test, salivary gland scintigraphy, or salivary flows).

## 3. Discussion

pSS is a chronic autoimmune disease characterized by infiltration of salivary glands, structural rearrangement, and expression of co-stimulatory molecules, cytokines, and chemokines. CCL2, also called monocyte chemotactic protein-1 (MCP-1), is a member of the C-C chemokine family that is involved in pSS. CCL2 is produced by many cell types, including endothelial, epithelial, smooth muscle, astrocytes, monocytic cells, fibroblasts, and microglial cells [47,48,49], and regulates the migration and infiltration of immune cell types such as monocytes and T lymphocytes. CCL2 may represent a potential intervention target for the treatment of various diseases, including autoimmune diseases [50,51]. Several studies demonstrated that CCL2 is highly expressed in the sera [52] and saliva [35,53] of pSS patients, especially in those with the presence of ectopic germinal structures in salivary gland biopsies [38,52]. In addition, in salivary glands of patients suffering from pSS, quantitative and qualitative abnormalities of saliva, altered localization of AQP5, and cell apoptosis might expose SGECs to chronic HOS rather than to transient HOS occurring under the physiological saliva-secretion process. Persistent HOS, which causes an osmotic efflux of water from the cell and an immediate shrinkage, is considered as a major risk for normal cell function and survival. The cellular ability to regulate the extracellular and intracellular solute environment is crucial for the maintenance of cell homeostasis and involves the activation of an osmoprotective program. Indeed, some cell types have the capacity to recover from HOS-induced cell volume modification by a so-called RVI mechanism. RVI involves immediate stimulation of Na^+^ and Cl^−^ ion influx followed by water influx and the delayed stimulation of NFTA5 that transactivates osmoprotective genes. NFAT5, originally identified as a tonicity-regulated factor involved in the cellular response to hyperosmotic stress, was also shown to stimulate the expression of proinflammatory cytokines in response to HOS, e.g., TNFα, IL-1β, IL-6, IL-8, and IL-18 [54]. 

Despite the chronic exposure of salivary gland epithelial cells to HOS in pSS, the effect of HOS on SGECs has received little attention. We have previously shown that the hyperosmolar environment of SGECs increased extracellular matrix remodeling and lymphocytic infiltration in pSS [41]. In addition, among the most 150 upregulated DEGs in NS-SV-AC cells common to NaCl and sucrose stimulation, we identified the C-C motif chemokine ligand 2 (CCL2) among a hub of genes corresponding to interferon (IFN)-induced genes [41]. 

Herein, we confirm the induction of CCL2 mRNA and protein levels upon NaCl stimulation of NS-SV-AC cells. In addition, we show that the mechanism involved in HOS-induced CCL2 transcription is independent of NFAT5 and NF-ĸB. Indeed, NFAT5-dominant negative plasmid does not abolish or significantly reduce the HOS-induced transcription of CCL2 in NS-SV-AC cells. Furthermore, considering the critical role of NF-ĸB in several inflammatory responses and its ability to bind to the CCL2 gene promoter [55,56], we also investigated the involvement of NF-ĸB in HOS-induced CCL2 transcription. Western blot analysis showed a strong activation of the canonical pathway of NF-ĸB under TNFα stimulation but not under Iso or Na100 treatment. In agreement with these data, the inhibition of NF-ĸB activation using Bay117085 abolished CCL2 transcription induced by TNFα but not by Na100. To further explore the mechanism involved in HOS-induced CCL2 transcription, we performed an oligonucleotide pulldown assay and identified KLF5 as a transcription factor binding differentially to the CCL2 gene promoter under both NaCl and sucrose stimulation. The role of KLF5 in CCL2 transcription was confirmed using one of its inhibitors (CID5951923) that decreased approximately by 27% the NaCl-induced CCL2 mRNA levels. Our data corroborate studies showing the involvement of KLF5 in the upregulation of CCL2 transcription in other cell types such as endothelial cells from an umbilical vein treated with TNFα [57] and microvessels treated with lipopolysaccharides [58]. However, our data are in opposition with some showing that KLF5 siRNA induced an upregulation of CCL2 mRNA levels in unstimulated human airway epithelial cells [59]. These data suggest a dichotomic effect of KLF5 on CCL2 transcription, acting either as an activator or repressor. This dichotomic effect of KLF5 may depend on the environmental/treatment conditions, and/or be cell specific. Indeed, more broadly, it has been well documented that KLF5 can switch from a transcriptional activator to a transcriptional repressor for the same sets of target genes upon post-translational modifications occurring downstream of environmental cues [60]. Considering that HOS activates p38 by phosphorylation [61] and that p38 activates KLF5 by phosphorylation [62], such a mechanism may therefore account for the HOS-driven CCL2 transcription observed in NS-SV-AC cells. However, additional studies will be required to substantiate this hypothesis. Results from our study do not exclude the participation of other transcription factors to the regulation of HOS-induced CCL2 transcription. Additional studies will be valuable to assess the binding site of KLF5 to the CCL2 gene promoter and to understand the mechanisms involved in the HOS-induced CCL2 transcription. 

From a pathophysiological point of view, our data suggest that chronic HOS could exacerbate pSS disease by contributing to the inflammatory process induced by the expression and secretion of CCL2. These observations are in agreement with studies performed on labial salivary gland biopsies from pSS patients showing in situ expression of CCL2 and CCR2 in the ductal structure and infiltrating mononuclear cells. In addition, primary SGECs from pSS in vitro produced a high level of CCL2 as observed by both ELISA and RT-PCR [53]. HOS-induced CCL2 expression and secretion may thus contribute to the recruitment of infiltrating immune cells. We also provided new data supporting the involvement of CCL2 in pSS pathogenesis and a relationship between CCL2 levels and the presence or absence of biological and subcutaneous manifestations and ESSDAI score. However, we have not observed any correlation between CCL2 and sicca symptoms, specifically xerostomia and salivary flow. Given our working hypothesis linking CCL2 production in salivary acinar cells to local hyperosmolarity due to glandular dysfunction, we anticipated observing a correlation between CCL2 levels and glandular dysfunction. However, several potential explanations arise, primarily stemming from the retrospective nature of this blood-sample-based study. First, xerostomia and dry eyes are subjective multifactorial complaints that may not necessarily correlate directly with the measurable activity and dysfunction of the glands in the disease. Moreover, the limited availability of salivary flow data for only a small subset of patients (cf. Table 1) may contribute to the absence of significant differences in this aspect. It is plausible that our study lacks the statistical power required to demonstrate a difference in salivary flow due to the limited sample size. Lastly, it is crucial to note that our measurements focus on serum CCL2 levels, which may not exclusively originate from salivary glands. This could explain why correlations are observed with certain systemic manifestations but not with items related to local salivary dysfunction. More extensive studies, using a larger cohort of well-documented patients, will be worthwhile to answer these questions.

Interferons and the interferons signature are well known for playing a role in autoimmune diseases, including in pSS [63]. In addition, CCL2 expression is induced by interferons [64,65,66], and HOS induces an interferon signature—including CCL2 [41]. Therefore, considering these observations, HOS may induce CCL2 expression via the release of interferons. Further studies will be necessary to explore this hypothesis.

In conclusion, our data support the role of KLF5 in HOS-induced CCL2 transcription while ruling out the involvement of NFAT5 and NF-kB and the involvement of CCL2 in pSS pathogenesis. 

## 4. Materials and Methods

### 4.1. Cell Culture and Treatment

Immortalized human salivary gland acinar cells, NS-SV-AC cells (a generous gift from Prof. M. Azuma, Second Department of Oral and Maxillofacial Surgery, Tokushima University School of Dentistry) [67], were grown in DMEM-HamF12 medium containing 5% heat-inactivated fetal calf serum, 100 UI/mL streptomycin-penicillin, and 4 mM glutamine (Thermo-Fisher Scientific, Walthman, MA, USA) and passaged twice a week. The cell line was tested free of mycoplasma contamination using the LookOut^®^ Mycoplasma PCR Detection Kit (Sigma-Aldrich, St. Louis, MO, USA). Cell line identity was confirmed by a short tandem repeat (STR) DNA profile (European Collection for Authenticated Cell cultures, Public Health England, UK). 

NS-SV-AC cells were seeded at 350,000 cells/well in 6 multi-well culture dishes and stimulated for 48 h post-transfection with iso-osmolar medium (Iso) or a medium containing an additional 100 mM of NaCl (Na100) or 200 mM of sucrose (Su200) or 25 ng/mL of TNFα for 8 h. For inhibitor treatment, NS-AV-AC cells were preincubated with different concentrations of inhibitors or DMSO and stimulated for 8 h with Iso, NaCl, and TNFα in the presence of DMSO or an inhibitor. For the determination of the CCL2 mRNA half-life, NS-AV-AC cells were incubated for 8 h with Iso, NaCl, and TNFα and then for various times in the presence of DMSO or 1 µM actinomycin D.

### 4.2. Cell Transfection and Reporter Assay

NS-SV-AC cells were transfected with NFAT-DN (a dominant negative form of NFAT5; generously donated from Ben C. B. Ko [68]), pSEAP-TonE (a reporter plasmid containing two TonE sites capable of binding the NFAT5 present upstream of the secreted alkaline phosphatase (SEAP) acting as the reporter gene (Clontech, Palo Alto, CA, USA); a generous gift from Christoph Küper [69]) and pGL4.32[luc2P/NF-ĸB-RE/Hygro] vector (pNFĸB-Luc; purchased from Promega (Madison, WI, USA) to reach a total of 7 µg per 2 × 10^6^ cells. Cells transfected with empty vector served as a negative control (CTN). Forty-eight hours post-transfection, cells were then subjected to treatments as described under Section 4.1.

### 4.3. Chemical Compounds

The Bay117085 purchased from Selleckchem (Houston, TX, USA) and CID5951923 from Tocris (Abingdon, UK) were used at 10 µM. 

### 4.4. Western Blot Analysis

Cells were harvested in lysis buffer (50 mM of HEPES, 150 mM of NaCl, 10 mM of EDTA, 10 mM of Na_4_P_2_O_7_^.^ 10 H_2_O, and a cocktail of proteases inhibitors), scraped and flash-frozen in liquid nitrogen. After thawing, the cell lysate was centrifuged for 20 min at 13,000× *g* at 4 °C, and the supernatant containing the total cell proteins was collected. Total cell protein concentration was assayed using the Pierce BCA protein assay (Thermo-Fischer Scientific, Waltham, MA, USA). Total proteins (20 µg/well) were separated by electrophoresis on a 12% or 8% SDS-PAGE Tris-glycine gel and then electrotransferred to a PVDF membrane (Invitrogen, Waltham, CA, USA). The PVDF membrane was blocked for 60 min at room temperature in 5% milk or 8% BSA diluted into PBS-0.1%Tween or TBS-0.1% Tween, according to the antibody’s provider instructions. Then, the PVDF membrane was incubated overnight at 4 °C with primary antibodies (1/1000 dilution of IKBα and phospho-NF-kB (Ser536); Cell signaling, Danvers, MA, USA) and then for 1 h at room temperature with anti-rabbit or anti-mouse HRP-conjugated antibody diluted at 1:3000 (Cell signaling, Danvers, MA, USA). Immunoblots were revealed by chemiluminescence (PerkinElmer, Waltham, MA, USA) using Kodak X-omat blue films or Amersham Imager 600 (GE Healthcare, Chicago, IL, USA).

### 4.5. RNA Extraction and cDNA Synthesis

Cellular RNA extraction, determination of both RNA concentration and purity, assessment of RNA integrity, and cDNA synthesis were carried out as previously described [70].

### 4.6. Quantitative Real-Time PCR

Primer’s design and quantitative real-time PCR (qRT-PCR) reactions were performed as previously described [70]. Data were analyzed using the Bio-Rad qPCR CFX system and normalized using selected appropriate stable reference genes (PPia, MDH1, and ATP5B) using Biogazelle qbase plus software (Biogazelle, Zwijnaarde, Belgium). All qRT-PCR were performed in agreement with the Minimal Information for Publication of Quantitative Real-time PCR Experiments (MIQE) guidelines [71]. Data are expressed as relative gene expression over control (set to 1) and are the mean ± S.E.M. of several independent experiments. 

### 4.7. Oligonucleotide Pulldown Assay

Six overlapping segments of 700 bp covering the CCL2 gene promoter were amplified by PCR using primers biotinylated at 3′ or 5′. Then, 5 µg of the mixed amplified sequences were incubated for 2 h at room temperature with 30 µL of streptavidin–agarose beads (Sigma-Aldrich, St. Louis, MI, USA). NS-SV-AC nuclear extract was prepared using the Nuclear Extract Kit (Active Motif, Carlsbad, CA, USA) according to the manufacturer’s instructions. Then, 400 µg of nuclear protein extract were incubated overnight at 4 °C with DNA–streptavidin–agarose beads. The complex was washed 2 times with PBS containing protease inhibitors and 3 times with a buffer containing 20 mM of TrisHCl and 2 mM of CaCl_2_. The nuclear proteins–DNA–streptavidin agarose beads complex was resuspended in 150 µL of the latter buffer and stored until further processing. 

### 4.8. Mass-Spectrometry Analysis

The nuclear proteins–DNA–streptavidin–agarose bead complexes were incubated for 4 h with 1 µg trypsin (Promega, Madison, WI, USA) at 37 °C. After removal of the beads, proteins were further digested overnight at 37 °C with 1 µg of trypsin. Peptides were purified on OMIX C18 tips (Agilent, Santa Clara, CA, USA), dried, and re-dissolved in 20 µL loading solvent A (0.1% trifluoroacetic acid in water/acetonitrile (ACN) (98:2, *v*/*v*)). Then, 5 µL were injected for LC-MS/MS analysis on an Ultimate 3000 RSLCnano system (Thermo-Fisher Scientific, Walthman, MA, USA) connected in line to a Q Exactive HF Biopharma mass spectrometer (Thermo-Fisher Scientific, Walthman, MA, USA). Trapping was performed at 10 μL/min for 4 min in loading solvent A on a 20 mm trapping column (made in-house, 100 μm internal diameter (I.D.), 5 μm beads, C18 Reprosil-HD, Dr. Maisch, Germany). The peptides were separated on a nanoEase column (MZ HSS T3 1.8 μm, 75 μm × 250 mm; Waters, Milford, MA, USA) kept at a constant temperature of 50 °C. Peptides were eluted by a nonlinear increase from 1 to 55% MS solvent B (0.1% FA in water/ACN (2:8, *v*/*v*)) over 65 min, at a flow rate of 300 nl/min, followed by a 5 min wash reaching 97% MS solvent B and re-equilibration with 99% MS solvent A (0.1% FA in water). The mass spectrometer was operated in data-dependent mode, automatically switching between MS and MS/MS acquisition for the 12 most abundant ion peaks per MS spectrum. Full-scan MS spectra (375–1500 *m*/*z*) were acquired at a resolution of 60,000 in the Orbitrap analyzer after accumulation to a target value of 3,000,000. The 12 most intense ions above a threshold value of 13,000 (minimum AGC of 1000) were isolated for fragmentation at a normalized collision energy of 30%. The C-trap was filled at a target value of 100,000 for a maximum of 80 ms, and the MS/MS spectra (200–2000 *m*/*z*) were acquired at a resolution of 15,000 in the Orbitrap analyzer with a fixed first mass of 145 *m*/*z*. Only peptides with charge states ranging from +2 to +6 were included for fragmentation, and the dynamic exclusion was set to 12 s. QCloud was used to control instrument longitudinal performance during the project [72]. Analysis of the mass spectrometry data was performed in MaxQuant (version 1.6.11.0) with mainly default search settings including a false discovery rate set at 1% on PSM, peptide, and protein level. Spectra were searched against the homo sapiens proteins in the Uniprot/Swiss-Prot database (database release version of January 2020 containing 20,365 human protein sequences, downloaded from http://www.uniprot.org). The mass tolerance for precursor and fragment ions was set to 4.5 and 20 ppm, respectively, during the main search. Enzyme specificity was set as C-terminal to arginine and lysine, also allowing cleavage at proline bonds with a maximum of two missed cleavages. Variable modifications were set to oxidation of methionine residues, acetylation of protein N-termini. Matching between runs was enabled with a matching time window of 0.7 min and an alignment time window of 20 min. Only proteins with at least one unique or razor peptide were retained leading to the identification of 2155 proteins. Proteins were quantified by the MaxLFQ algorithm integrated in the MaxQuant software (version 1.6.11.0). A minimum ratio count of two unique or razor peptides was required for quantification. Further data analysis was performed with the Perseus software (version 1.6.2.1) after loading the protein groups file from MaxQuant. Reverse database hits were removed, label-free quantitation (LFQ) intensities were log2 transformed, and replicate samples were grouped. Proteins with fewer than three valid values in at least one group were removed and missing values were imputed from a normal distribution around the detection limit leading to a list of 1611 quantified proteins that was used for further data analysis. A *t*-test was performed (FDR = 0.05) to compare stimulated conditions (Na100 or Su200 or TNFα) to Iso condition prior to generating volcano plots. 

### 4.9. Patients

Sera samples from 50 pSS patients fulfilling the American College of Rheumatology (ACR)/European League against Rheumatism (EULAR) classification criteria were used for CCL2 Milliplex© MAP KIT #HCYTMAG-60K (EDM Millipore, Billerica, MA, USA). All studies were conducted in agreement with the ULB Erasme Hospital ethics committee (Protocols P2020/128 and P2020/351). Patient characteristics are summarized in Table 1.

### 4.10. CCL2 ELISA

CCL2 protein levels were determined using an CCL2 ELISA kit (Merck-Millipore, Burlington, MA, USA). 

### 4.11. Statistical Analysis

Statistical analyses were performed using GraphPad Prism 10.0.3 (New York, NY, USA) and repeated-measures Anova followed by post hoc Dunnett tests, two-way repeated-measures Anova, paired Student *t*-test, *t*-test for unique samples, Spearman’s r, and Mann–Whitney’s U-test. The tests were considered statistically significant when the *p*-value was <0.05. Proteomic data were analyzed using the Benjamini Hochberg *t*-test, and results were considered statistically significant when the false discovery rate (FDR) was <0.05. 

## Figures and Tables

**Table 1:** Patient characteristics.

Demographic Data		
Age; N = 50	Mean ± SD	55.47 ± 13.1
Female	n/N (%)	45/50 (90%)
Current tobacco smoking	n/N (%)	5/36 (13.9%)
Focus score; N = 45	Median [range]	1.28 [0;12]
**Sicca-Asthenia-Polyalgia triad**		
ESSPRI (x/100); N = 11	Mean ± SD	61.81 ± 27.1
Fatigue	n/N (%)	29/46 (63%)
Widespread pain	n/N (%)	27/48 (56.3%)
Xerostomia	n/N (%)	47/49 (95.9%)
Dry eyes complaints	n/N (%)	43/49 (87.8%)
Abnormal Schirmer’s test	n/N (%)	30/38 (79%)
Abnormal salivary scintigraphy	n/N (%)	12/21 (57.1%)
Salivary flow rates (mL/min)		
Unstimulated salivary flow; N = 13	Median [range]	0.1 [0.01;0.5]
Stimulated salivary flow rate (Saxon test); N = 9	Mean ± SD	1.50 ± 0.54
Raynaud phenomenon	n/N (%)	10/30 (33.3%)
**Serological Biomarkers**		
Rheumatoid factor	n/N (%)	19/46 (41.3%)
Anti-CCP	n/N (%)	0/26 (0%)
Antinuclear antibodies		
Positivity	n/N (%)	36/50 (72%)
Titer (1/x); N = 36	Median [range]	240 [80;5000]
Speckled pattern	n/N (%)	34/50 (68%)—94% of ANA+
Anti-SSA/Ro60 positivity	n/N (%)	30/44 (68.2%)
Anti-SSA/Ro52 positivity	n/N (%)	15/34 (44.12%)
Anti-SSB/La positivity	n/N (%)	12/45 (26.7%)
C4 consumption	n/N (%)	3/47 (6.4%)
Cryoglobulinemia	n/N (%)	2/36 (5.56%)
Beta2-microglobulin (mg/L); N = 29	Median [range]	2 [1.25;4.4]
Hypergammaglobulinemia	n/N (%)	15/49 (30.61%)
Monoclonal component	n/N (%)	14/47 (29.8%)
**Intention to treat (after sampling)**		
Corticoids	n/N (%)	10/50 (20%)
Hydroxychloroquine	n/N (%)	10/50 (20%)
Immunosuppressant	n/N (%)	9/50 (18%)
**Disease activity (as defined in ESSDAI score)**		
ESSDAI; N = 50	Median [range]	2 [0;28]
pSS with systemic manifestations (ESSDAI > =5); N = 50	n/N (%)	18/50 (36%)
Constitutional	n/N (%)	2/50 (4%)
Lymphadenopathy	n/N (%)	4/50 (8%)

n/N: number of patients with specific item (n) over total number of patients (N).

## Data Availability

Authors agree to make data and materials supporting the results or analyses presented in their paper available upon reasonable request.
